# In vivo white matter microstructure in adolescents with early-onset psychosis: a multi-site mega-analysis

**DOI:** 10.1038/s41380-022-01901-3

**Published:** 2022-12-12

**Authors:** Claudia Barth, Sinead Kelly, Stener Nerland, Neda Jahanshad, Clara Alloza, Sonia Ambrogi, Ole A. Andreassen, Dimitrios Andreou, Celso Arango, Inmaculada Baeza, Nerisa Banaj, Carrie E. Bearden, Michael Berk, Hannes Bohman, Josefina Castro-Fornieles, Yann Chye, Benedicto Crespo-Facorro, Elena de la Serna, Covadonga M. Díaz-Caneja, Tiril P. Gurholt, Catherine E. Hegarty, Anthony James, Joost Janssen, Cecilie Johannessen, Erik G. Jönsson, Katherine H. Karlsgodt, Peter Kochunov, Noemi G. Lois, Mathias Lundberg, Anne M. Myhre, Saül Pascual-Diaz, Fabrizio Piras, Runar E. Smelror, Gianfranco Spalletta, Therese S. Stokkan, Gisela Sugranyes, Chao Suo, Sophia I. Thomopoulos, Diana Tordesillas-Gutiérrez, Daniela Vecchio, Kirsten Wedervang-Resell, Laura A. Wortinger, Paul M. Thompson, Ingrid Agartz

**Affiliations:** 1 Department of Psychiatric Research, Diakonhjemmet Hospital, Oslo, Norway; 2 Norwegian Centre for Mental Disorders Research (NORMENT), Institute of Clinical Medicine, University of Oslo, Oslo, Norway; 3 Department of Psychosis Studies, King’s College London, London, UK; 4 Imaging Genetics Center, Mark & Mary Stevens Neuroimaging & Informatics Institute, Keck School of Medicine, University of Southern California, Marina del Rey, CA USA; 5 Department of Child and Adolescent Psychiatry, Institute of Psychiatry and Mental Health, Hospital General Universitario Gregorio Marañón, IiSGM, CIBERSAM, Madrid, Spain; 6 Laboratory of Neuropsychiatry, Santa Lucia Foundation IRCCS, Rome, Italy; 7 Norwegian Center for Mental Disorders Research (NORMENT), Division of Mental Health and Addiction, Oslo University Hospital, Oslo, Norway; 8 Centre for Psychiatry Research, Department of Clinical Neuroscience, Karolinska Institutet & Stockholm Health Care Services, Stockholm Region, Stockholm, Sweden; 9 School of Medicine, Universidad Complutense, Madrid, Spain; 10 Department Child and Adolescent Psychiatry and Psychology, 2017SGR881 Institute of Neuroscience, Hospital Clinic Barcelona. CIBERSAM. August Pi i Sunyer Biomedical Research Institute (IDIBAPS), University of Barcelona, Barcelona, Spain; 11 Department of Psychiatry and Biobehavioral Sciences, Semel Institute for Neuroscience and Human Behavior, UCLA, Los Angeles, CA USA; 12 Department of Psychology, UCLA, Los Angeles, CA USA; 13 Deakin University, Institute for Mental and Physical Health and Clinical Translation, School of Medicine, Barwon Health, Geelong, Australia; 14 Department of Neuroscience, Child and Adolescent Psychiatry, Uppsala University, Uppsala, Sweden; 15 Department of Clinical Science and Education Södersjukhuset, Karolinska Institutet, Stockholm, Sweden; 16 Turner Institute for Brain and Mental Health and School of Psychological Sciences, Monash University, Melbourne, VIC Australia; 17 Hospital Universitario Virgen del Rocío, Universidad de Sevilla, Department of Psychiatry, CIBERSAM, IBiS-CSIC, Sevilla, Spain; 18 Highfield Unit, Warneford Hospital, Oxford, UK; 19 Department of Psychiatry, University of Oxford, Oxford, UK; 20 Maryland Psychiatric Research Center, Department of Psychiatry, University of Maryland School of Medicine, Baltimore, MA USA; 21 Department of Child and Adolescent Psychiatry, Institute of Psychiatry and Mental Health, Hospital General Universitario Gregorio Marañón, IiSGM, Madrid, Spain; 22 Section of Child and Adolescent Mental Health Research, Division of Mental Health and Addiction, Oslo University Hospital, Oslo, Norway; 23 Magnetic Resonance Imaging Core Facility, August Pi i Sunyer Biomedical Research Institute (IDIBAPS), University of Barcelona, Barcelona, Spain; 24 Menninger Department of Psychiatry and Behavioral Sciences, Baylor College of Medicine, Houston, TX USA; 25 Department of Radiology, Marqués de Valdecilla University Hospital, Valdecilla Biomedical Research Institute IDIVAL, Santander (Cantabria), Spain; 26 Advanced Computing and e-Science, Instituto de Física de Cantabria (UC-CSIC), Santander (Cantabria), Spain

**Keywords:** Schizophrenia, Bipolar disorder, Neuroscience

## Abstract

Emerging evidence suggests brain white matter alterations in adolescents with early-onset psychosis (EOP; age of onset <18 years). However, as neuroimaging methods vary and sample sizes are modest, results remain inconclusive. Using harmonized data processing protocols and a mega-analytic approach, we compared white matter microstructure in EOP and healthy controls using diffusion tensor imaging (DTI). Our sample included 321 adolescents with EOP (median age = 16.6 years, interquartile range (IQR) = 2.14, 46.4% females) and 265 adolescent healthy controls (median age = 16.2 years, IQR = 2.43, 57.7% females) pooled from nine sites. All sites extracted mean fractional anisotropy (FA), mean diffusivity (MD), radial diffusivity (RD), and axial diffusivity (AD) for 25 white matter regions of interest per participant. ComBat harmonization was performed for all DTI measures to adjust for scanner differences. Multiple linear regression models were fitted to investigate case-control differences and associations with clinical variables in regional DTI measures. We found widespread lower FA in EOP compared to healthy controls, with the largest effect sizes in the superior longitudinal fasciculus (Cohen’s *d* = 0.37), posterior corona radiata (*d* = 0.32), and superior fronto‐occipital fasciculus (*d* = 0.31). We also found widespread higher RD and more localized higher MD and AD. We detected significant effects of diagnostic subgroup, sex, and duration of illness, but not medication status. Using the largest EOP DTI sample to date, our findings suggest a profile of widespread white matter microstructure alterations in adolescents with EOP, most prominently in male individuals with early-onset schizophrenia and individuals with a shorter duration of illness.

## Introduction

Brain white matter alterations are well-documented in adults with psychotic disorders. A recent meta-analysis from the Enhancing Neuro Imaging Genetics through Meta-Analysis (ENIGMA) Consortium (*n* = 4322) reported widespread lower fractional anisotropy (FA) in adult individuals with schizophrenia relative to healthy controls, with the largest effect sizes in the anterior corona radiata (*d* = 0.40) and corpus callosum (*d* = 0.39) [1]. Emerging evidence suggests similar alterations in adolescents with early-onset psychosis (EOP). However, as neuroimaging methods vary across studies and sample sizes are small to modest, results in EOP remain inconclusive [2]. To address this issue, the ENIGMA EOP Working Group initiated the largest collaborative mega-analysis of white matter microstructure in EOP to date.

The term EOP covers rare and heterogeneous psychotic disorders, affecting 0.05–0.5% of the world’s population [3, 4], and encompasses both the schizophrenia and affective psychosis spectra. Compared to adult-onset psychosis, individuals with EOP show worse long-term prognosis [5–8], and EOP significantly contributes to the lifetime disease burden for adolescents [9]. Psychotic symptoms in EOP emerge before 18 years of age, during adolescence [10]—a sensitive period for brain development. To date, there is insufficient knowledge on how brain maturation is linked to the emergence of psychosis and neuroimaging studies on these co-occurring processes are important. While a few magnetic resonance imaging (MRI) studies in EOP report grey matter brain abnormalities [11–15], less is known about putative white matter alterations. This is a critical research gap, as understanding how white matter microstructure is affected in EOP may provide important insights into the pathophysiology of psychotic disorders during adolescent brain development.

Microstructural properties of white matter are commonly modelled using diffusion tensor imaging (DTI), which maps the Brownian movement of water molecules in the brain in vivo. Common DTI measures include FA and mean, axial, and radial diffusivity (MD, AD, RD). While FA is a summary measure that reflects the degree of diffusion directionality, AD describes diffusion along the primary axis, and RD characterizes diffusion perpendicular to it [16]. MD is a measure of overall diffusion within a voxel. Although FA is generally sensitive to microstructural changes, it is not specific to the type of change (e.g., radial or axial) [16]. Both AD and RD have been associated with different putative biological underpinnings: lower AD has been linked to axonal damage [16] and higher RD to disruptions in myelination [17]. These DTI measures change throughout the lifespan, with FA increasing and RD and MD decreasing throughout adolescence until early adulthood in healthy individuals [18, 19]. Sex differences in this pattern also exist: females show changes in white matter microstructure mainly during mid-adolescence, while white matter changes in males appear to occur from childhood through early adulthood [20]. The trajectories of AD are less well known [18, 19].

Several studies have used DTI to compare white matter microstructure in youth with EOP to healthy controls, predominantly focusing on early-onset schizophrenia (EOS [2]). Most studies found widespread lower FA in individuals with EOP. However, the white matter tracts implicated were highly variable across studies [21–26]. Common DTI measures beyond FA have rarely been explored. The low degree of spatial overlap between the studies may stem from phenotypic heterogeneity, such as differences in disease severity and duration, medication history, and comorbidities. Small sample sizes and differences in MRI data acquisition, processing, and analysis may further influence study outcomes.

The ENIGMA-EOP Working Group aims to address some of the methodological issues in prior MRI studies and increase statistical power by pooling data for the largest coordinated analysis on brain white matter in EOP to date [27]. The primary goal of the present study was to identify white matter differences in EOP relative to healthy controls using a mega-analytic approach [28]. We further included a complementary meta-analysis to illustrate between-cohort heterogeneity and allow for a direct comparison to FA findings in adult individuals with schizophrenia [1]. The modulating effects of sex and clinical covariates such as medication use, symptom severity, and illness duration on DTI measures were also investigated. Based on previous findings [2], we hypothesized that adolescents with EOP would show widespread lower FA relative to healthy controls. As consistent evidence for associations between DTI measures and clinical covariates in EOP is lacking [2], follow-up analyses were exploratory in nature.

## Materials & methods

### Study sample

The ENIGMA-EOP Working Group obtained case-control data from nine cohorts across seven countries (for information on each cohort, please see Supplementary Table S1, S2, and S3), yielding imaging and clinical data on a combined total of 321 adolescents with early-onset psychosis (EOP) and 265 age-matched healthy controls. Participants were aged 12 to 18 years at MRI image acquisition. The EOP diagnostic subgroups consisted of individuals with early-onset schizophrenia (EOS; *n* = 180), affective psychosis (AFP; *n* = 95), and other psychosis (*n* = 46). Diagnoses were determined using either the Diagnostic and Statistical Manual of Mental Disorders (DSM)-IV or the International Classification of Diseases (ICD)−10. To assess the presence and severity of symptoms, five cohorts used the Positive and Negative Syndrome Scale (PANSS [29]), and two cohorts used the Scale for the Assessment of Negative/Positive Symptoms (SANS [30]/SAPS [31]). Two cohorts did not acquire PANSS, SANS/SAPS or equivalent scores. Site-wise inclusion and exclusion criteria are presented in Supplementary Table S2. All study participants and/or their legal guardians provided written informed consent with approval from local institutional review boards and the respective ethics committees. The study was conducted in accordance with the Declaration of Helsinki.

### Image processing and analysis

MRI scanner and acquisition parameters for each site are detailed in Supplementary Table S3. Preprocessing of diffusion-weighted images, including eddy current correction, echo-planar imaging induced distortion correction, initial quality control, and tensor fitting were performed locally at each site using tools and processes suitable for the acquired data. As head motion can be a confound in DWI studies [32], we tested for case-control differences in motion parameters by site and scanner, based on the outputs of the eddy current correction. No significant differences were found (Table S4). Protocols for image processing and quality control procedures are available via the ENIGMA-DTI website (http://enigma.ini.usc.edu/ongoing/dti-working-group/) and on the ENIGMA GitHub page (https://github.com/ENIGMA-git/). Fractional anisotropy (FA), mean diffusivity (MD), radial diffusivity (RD), and axial diffusivity (AD) were obtained for 25 bilateral (or mid-sagittal) regions of interest (ROI) from the Johns Hopkins University ICBM-DTI-81 white-matter labels atlas (JHU;[33] see Table 1 for a list of white matter regions). While all nine sites provided FA measures (*n* = 586), MD, RD, and AD data were obtained from eight sites (*n* = 505, see Supplementary Note S1).

**Table 1:** Twenty-five white matter regions of interest.

Brain white matter tract	Abbreviation
Average DTI measure across entire skeleton	Average FA/MD/RD/AD
Corpus callosum (body/genu/splenium)	CC/BCC/GCC/SCC
Cingulum (cingulate gyrus part)	CGC
Perihippocampal cingulum tract	CGH
Corona radiata (anterior/posterior/superior)	CR/ACR/PCR/SCR
Cortico‐spinal tract	CST
External capsule	EC
Fornix/Stria terminalis	FX/ FXST
Internal capsule (anterior/posterior/retrolenticular limb)	IC/ALIC/PLIC/RLIC
Inferior fronto-occipital fasciculus	IFO
Uncinate fasciculus	UNC
Posterior thalamic radiation	PTR
Superior fronto‐occipital fasciculus	SFO
Superior longitudinal fasciculus	SLF
Sagittal stratum	SS

ComBat harmonization was performed for all DTI measures to remove unwanted scanner- and sequence-related variation [34, 35] whilst preserving biological associations in the data (Supplementary Note S2). Empirical Bayes was used to leverage information across each DTI measure, an approach that has been shown to be more robust to outliers of small within-scanner sample sizes [36]. Age, sex, and diagnostic group were included as variables of interest. The ComBat-harmonization output for all DTI measures is visualized in Figs. S1 and S2.

### Statistical analysis

#### Case-control findings

Case-control differences in FA, MD, AD, and RD across all ROIs were examined using multiple linear regression analysis, correcting for age, age [2], sex, and linear age-by-sex and nonlinear age [2]-by-sex interactions. All analyses included combined ROIs across both hemispheres as dependent variables. Lateralized results are reported in Supplementary Table S9. We included a complementary meta-analysis to illustrate between-cohort heterogeneity [15] and allow for a direct comparison to FA findings in adult individuals with schizophrenia (see Supplementary Note S3 and section “*Comparison to adult individuals with schizophrenia”*).

#### Controlling for average, core, and periphery diffusivity measures

To examine global vs. regional white matter effects between diagnostic groups, the main DTI analyses were re-run either co-varying for (i) average, (ii) core, and (iii) periphery DTI measures. For instance, average FA constitutes FA averaged across the entire white matter skeleton, excluding gray matter. However, average FA is not only comprised of the JHU atlas regions, but also FA in white matter outside of these regions. Therefore, we separately calculated average FA for the “core”, which is defined as the region within the skeleton labeled by the JHU white matter atlas, and the “periphery”, outside of the JHU atlas regions. The average FA in the standard ENIGMA-DTI template consists of 112,889 voxels, while the core consists of 31,742 voxels, less than a third of the average FA. The remaining 81,147 voxels surrounding the core comprise the periphery (non-JHU). Detailed formulas to calculate core and periphery DTI measures have been published in Kelly et al. 2018 [1]. A figure displaying the difference in average, core, and periphery FA can be found in the supplemental materials (Fig. S3).

#### Diagnostic subgroup analysis

For each DTI measure (FA, MD, RD, AD) as dependent variable, separate multiple linear regression models were fitted with diagnostic subgroup as fixed factor (healthy controls, EOS, AFP, other psychosis) and age, age [2], sex, and linear age-by-sex and nonlinear age [2]-by-sex interactions as covariates.

#### Sex-by-diagnosis & age-by-diagnosis interactions

To explore white matter microstructural differences between diagnostic groups across age and sex, we performed follow-up sex-stratified as well as sex-by-diagnosis and age-by-diagnosis interaction analyses. Separate multiple linear regression models for each DTI measure as dependent variable were fitted either including a sex-by-diagnosis or age-by-diagnosis interaction terms. The same covariates as above apply. The sex-stratified case-control models (female only/ males only) were covaried for age and age [2].

#### Associations with medication and other clinical measures

In adolescents with EOP, we tested for effects of duration of illness, age at onset, PANSS scores (negative/positive subscores), current medication use (user vs. non-user) and antipsychotic chlorpromazine equivalents (CPZ, see Woods 2005 http://www.scottwilliamwoods.com/files/Equivtext.doc) on DTI measures. Current medication use included antipsychotics, lithium, antidepressants, and antiepileptics. As age and age at onset are highly correlated (*r* = 0.70), this linear model was only adjusted for sex, whereas the other models were adjusted for age, age [2], sex, and linear age-by-sex and nonlinear age [2]-by-sex interactions.

#### Comparison to adult individuals with schizophrenia

To assess whether the effect sizes of the case-control differences in tract-specific FA differed between adolescents with EOP and adults with schizophrenia [1], we conducted z-tests using the following formula [37]:

$$Diff = M_B - M_A$$

$$SE_{Diff} = \sqrt {V_{MA} + V_{MB}}$$

$$Z_{Diff} = \frac{{Diff}}{{SE_{Diff}}}$$

where M_A_ and M_B_ are the estimated Cohen’s *d* effect sizes of the schizophrenia and EOP sample, respectively. V_MA_ and V_MB_ reflect their variances as standard error (SE). The corresponding *p*-value was calculated using (two-tailed test):

$$p = 2[1 - (\Phi \left( {\left| {Z_{Diff}} \right|} \right))]$$

where Φ(Z) is the standard normal cumulative distribution. Meta-analytically derived Cohen’s *d* values for the adult schizophrenia sample were retrieved by SK [1] and compared to meta- and mega-analytically derived Cohen’s *d* values for EOP (adolescent sample, n=586; adult sample, *n* = 4322). To allow for such a comparison, the same covariates were used in both studies, namely age, age [2], sex, and linear age-by-sex and nonlinear age [2]-by-sex interactions.

To further test whether brain alterations in EOP resemble the pattern observed in adult schizophrenia, we correlated the meta- and mega-analytically derived Cohen’s *d* effect sizes for tract-specific FA from the current study with the meta-analytically derived Cohen’s *d* values from the adult schizophrenia study [38].

All statistical tests were conducted in R, version 4.1.0 (https://www.R-project.org/). All continuous variables were mean-centered before entered into the analyses. We computed the Cohen’s d effect sizes ± standard error across all 25 ROIs from the t-statistics for categorical variables [39]. To control for multiple comparisons, effects were considered significant if they survived the Bonferroni correction threshold of 0.05/25 = 0.002. All data and code produced in the present study are available upon reasonable request to the authors.

## Results

### Demographic and clinical variables

Demographics and clinical characteristics for the whole sample and stratified by diagnostic subgroups are summarized in Table 2 and Table 3, respectively. Sample measures stratified by cohort and sex are displayed in Table S5 and Table S6, respectively.

**Table 2:** Sample demographics and clinical measures.

Variables	CTR	EOP	*p*-value	Test
**N**	265	321		
**Age** (years)*	16.18 [14.88, 17.31]	16.57 [15.18, 17.32]	0.147	KW
**Sex**, female *N* (%)	153 (57.7)	149 (46.4)	**0.008**	χ2
**Handedness**, *N* (%)			0.331	χ2
*Right*	177 (90.8)	166 (88.8)		
*Left*	18 (9.2)	19 (10.2)		
*Ambidextrous*	0 (0.0)	2 (1.1)		
**Diagnostic subgroup**, *N* (%)				
*EOS*		180 (56.1)		
*AFP*		95 (29.6)		
*OTP*		46 (14.3)		
**PANSS**, negative*		16.00 [12.00, 21.00]		
**PANSS**, positive*		20.00 [16.00, 24.00]		
**Age of onset** (years)*		15.43 [14.12, 16.71]		
**Duration of illness** (years)*		0.60 [0.12, 1.10]		
CPZ*		200.0 [133.3, 333.3]		
**AP user**, *N* (%)		256 (89.8)		
**Lithium user**, *N* (%)		27 (9.9)		
**AD user**, *N* (%)		57 (25.0)		
**AE user**, *N* (%)		14 (5.2)		
**Field strength**, 3T, *N* (%)	95 (55.2)	153 (60.5)	0.329	χ2

*Non-normal continuous data in median [Interquartile range] and categorical data as number (%). Across diagnostic groups, we examined whether variables were normally distributed using Shapiro-Wilk tests and histograms. *CTR* healthy controls, *EOP* early-onset psychosis, *N* number, *EOS* early-onset schizophrenia, *AFP* affective psychosis, *OTP* other psychosis, *PANSS* positive and negative syndrome scale, *CPZ* chlorpromazine equivalent, *AP* antipsychotics, *AD* antidepressants, *AE* antiepileptics, *KW* Kruskal-Wallis. Significant results are highlighted in bold.

**Table 3:** Demographics and clinical measures, EOP stratified by diagnostic subgroups.

Variables	CTR	EOS	AFP	OTP	*p*-value general	*p*-value EOS vs. AFP	*p*-value EOS vs. OTP	*p*-value AFP vs. OTP	test
***N***	265	180	95	46					
**Age** (years)*	16.18 [14.88, 17.31]	16.59 [15.39, 17.25]	16.66 [15.41, 17.39]	15.85 [15.00, 17.38]	0.305	0.497	0.327	0.258	KW
**Sex**, female *N* (%)	153 (57.7)	70 (38.9)	54 (56.8)	25 (54.3)	**0.001**	**0.007**	0.084	0.921	χ2
**Handedness**, *N* (%)					0.564	0.773	0.510	0.957	χ2
Right	177 (90.8)	120 (89.6)	24 (88.9)	22 (84.6)					
Left	18 (9.2)	12 (9.0)	3 (11.1)	4 (15.4)					
Ambidextrous	0 (0.0)	2 (1.5)	0 (0.0)	0 (0.0)					
**PANSS**, negative*		18.00 [14.00, 23.00]	14.00 [10.00, 19.50]	15.00 [11.00, 18.00]	**<0.001**	**<0.001**	**0.001**	0.640	KW
**PANSS**, positive*		21.00 [18.00, 24.00]	19.00 [13.00, 24.00]	16.00 [14.00, 22.00]	**0.003**	**0.033**	**0.001**	0.293	KW
**Age of onset** (years)*		15.40 [14.00, 16.44]	16.05 [14.64, 16.94]	15.00 [14.20, 16.64]	**0.007**	**0.002**	0.912	0.056	KW
**Duration of illness** (years)*		1.00 [0.24, 1.49]	0.21 [0.08, 0.57]	0.62 [0.22, 1.04]	**<0.001**	**<0.001**	0.381	**0.003**	KW
CPZ*		200.0 [150.0, 383.5]	183.3 [100.0, 300.0]	166.7 [100.0, 258.3]	**0.033**	**0.033**	**0.042**	0.648	KW
**AP user**, *N* (%)		156 (90.2)	71 (89.9)	29 (87.9)	0.923	1.000	0.932	1.000	χ2
**Lithium user**, *N* (%)		3 (1.8)	24 (32.9)	0 (0.0)	**<0.001**	**<0.001**	1.000	**0.001**	χ2
**AD user**, *N* (%)		18 (14.4)	34 (47.9)	5 (15.6)	**<0.001**	**<0.001**	1.000	**0.004**	χ2
**AE user**, *N* (%)		11 (6.5)	3 (4.2)	0 (0.0)	0.283	0.691	0.286	0.584	χ2
**Field strength**, 3T, *N* (%)	95 (55.2)	69 (43.7)	65 (89.0)	19 (86.4)	**<0.001**	**<0.001**	**0.001**	1.000	χ2

*Non-normal continuous data in median [Interquartile range] and categorical data as number (%). Across diagnostic groups, we examined whether variables were normally distributed using Shapiro-Wilk tests and histograms. *CTR* healthy controls, *EOP* early-onset psychosis, *EOS* early-onset schizophrenia, *AFP* affective psychosis, *OTP* other psychosis, *N* number, *PANSS* positive and negative syndrome scale, *CPZ* chlorpromazine equivalent, *AP* antipsychotics, *AD* antidepressants, *AE* antiepileptics, *KW* Kruskal-Wallis. Significant results are highlighted in bold.

### Case-control differences

**Fig. 1:**
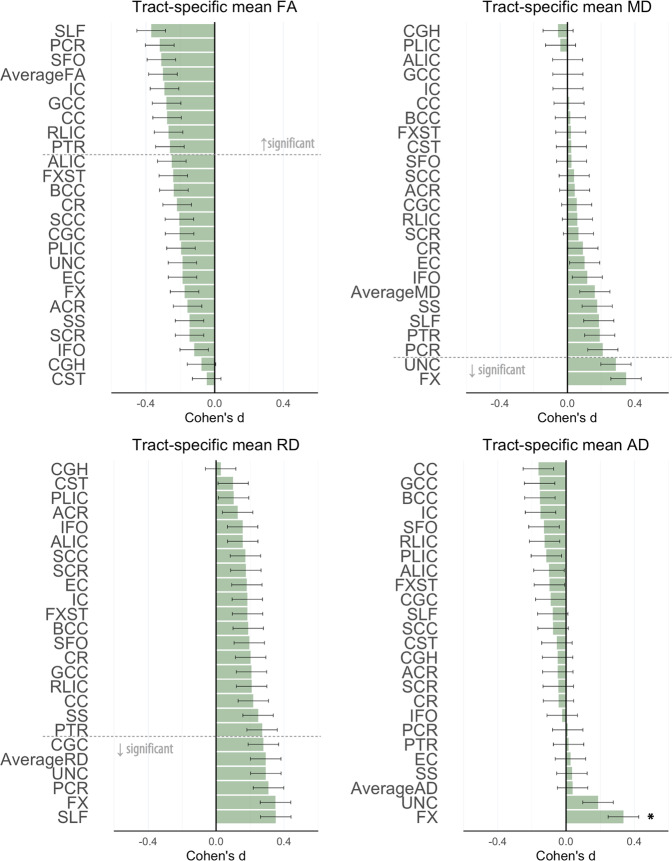
Cohen’s *d* values for differences in diffusion measures between adolescents with early-onset psychosis and healthy controls. Cohen’s *d* values and their standard errors are displayed, sorted by effect size. Stars and dashed lines indicate significant results (*p* ≤ 0.002). *FA* fractional anisotropy, MD mean diffusivity, RD radial diffusivity, AD axial diffusivity. For white matter tract abbreviations, see Table 1.

The mega‐analysis revealed widespread lower FA in adolescents with EOP relative to healthy controls (see Fig. 1, Supplementary Table S7), including the Average FA, CC, GCC, IC, PCR, PTR, RLIC, SFO, and SLF (*p* ≤ 0.002). Follow-up analyses showed higher MD in the FX and UNC; higher RD in Average RD, CGC, FX, PCR, SLF, and UNC; and higher AD in the FX in adolescents with EOP relative to healthy controls (*p* ≤ 0.002, see Fig. 1).

The complementary meta-analysis of case-control FA differences corroborated the significant effect for the SLF (Supplementary Table S16). Forest plots illustrate the variability among sites (Supplementary Fig. 8), suggesting a great degree of heterogeneity. However, a post hoc leave-one-out analysis revealed that no individual site had an influential impact on the significant finding (see Supplementary Note S3, Fig. S9, Table S17).

### Controlling for average, core, and periphery diffusivity measures

Adjusting for average FA or core FA, none of the FA case-control differences remained significant. Covarying for periphery FA, there was significantly lower FA in the SLF only (*p* ≤ 0.002, see Supplementary Table S8 and Fig. S4–7). For MD, after covarying for average, core, or periphery MD, the significance of case-control differences in the FX remained. For RD, no case-control differences remained significant after adjustment for average, core, or periphery RD. Lower AD in the FX remained significant after all additional adjustments.

### Diagnostic subgroup findings

**Fig. 2:**
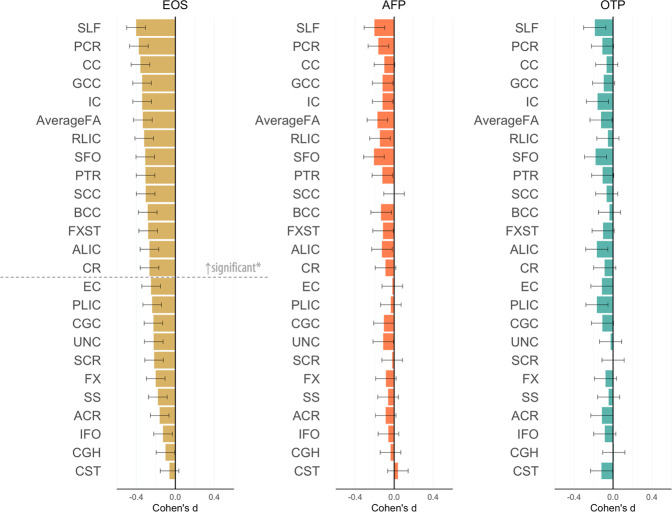
Fractional anisotropy (FA) differences between adolescents with early-onset psychosis and healthy controls, stratified by diagnostic subgroups. Cohen’s d values and their standard errors are displayed, sorted by effect size for EOS. Stars and dashed lines indicate significant results (*p* ≤ 0.002). Abbreviations: EOS = early-onset schizophrenia (*n* = 180), AFP affective psychosis (*n* = 95), OTP other psychosis (*n* = 46). For white matter tract abbreviations see Table 1.

When stratifying EOP by diagnostic subgroup, only adolescents with EOS showed significantly lower FA in 14 ROIs relative to healthy controls, including: Average FA, ALIC, BCC, CC, CR, FXST, GCC, IC, PCR, PTR, RLIC, SCC, SFO and SLF (*p* ≤ 0.002; see Fig. 2 and Supplementary Table S11). MD and AD were only significantly higher in the FX of adolescents with EOS compared to healthy controls. We found higher RD in the CC, CGC, FX, PCR, PTR, RLIC, SLF, and SS in adolescents with EOS, and higher Average RD in adolescents with AFP, relative to healthy controls (*p* ≤ 0.002). We observed no significant white matter microstructural alterations in adolescents with other psychosis relative to healthy controls.

### Sex-by-diagnosis and age-by-diagnosis interactions

**Fig. 3:**
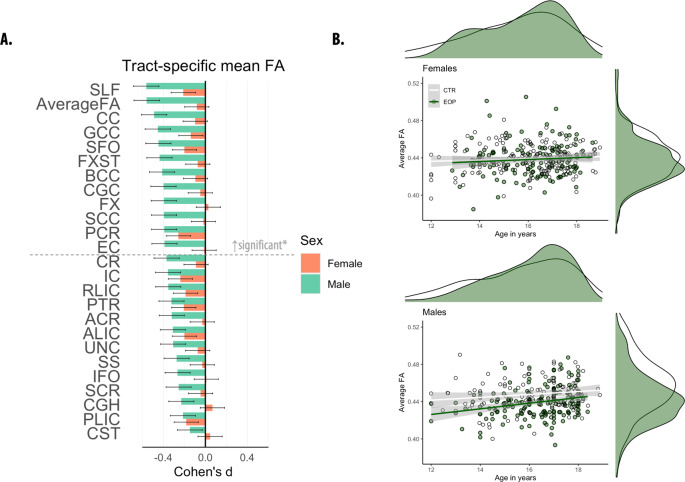
Fractional anisotropy (FA) differences between adolescents with early-onset psychosis and healthy controls, stratified by sex. **A** Cohen’s *d* values and their standard errors are displayed, sorted by effect size for males. Stars and dashed lines indicate significant results (*p* ≤ 0.002) for males only*. In females, FA did not differ significantly between individuals with EOP and healthy controls. For white matter tract abbreviations see Table 1. **B** Marginal plots with distributions displaying average FA across the entire skeleton and age for females (upper panel) and males (lower panel) by diagnostic group. Diagnostic group-specific regression lines are shown.

To examine sex differences in relation to diagnosis, sex-by-diagnosis interactions were estimated. After Bonferroni correction, no significant interactions for FA, MD, and AD were found (Supplementary Table S12). We did find a significant sex-by-diagnosis interaction for RD in the SCC (*p* ≤ 0.002). Sex-stratified analyses showed that only male adolescents with EOP showed widespread lower FA relative to healthy male controls (EOP = 172, healthy controls = 112; *p* ≤ 0.002, 12 ROIs: Average FA, BCC, CC, CGC, EC, FX, FXST, GCC, PCR, SCC, SFO, and SLF; Fig. 3, Supplementary Table S10). Similarly, tract-specific RD and MD were only higher in males with EOP relative to healthy male controls (*p* ≤ 0.002, Males: EOP = 147, healthy controls = 98, *RD*: Average RD, CC, CGC, CR, FX, FXST, PCR, PTR, SCC, SLF, SS, UNC; *MD*: PCR). Tract-specific AD was not significantly different in EOP vs. healthy controls. In females, FA, MD, RD and AD did not differ significantly between EOP and healthy controls after correction for multiple comparisons (FA: EOP = 149, healthy controls = 153; non-FA: EOP = 131, healthy controls = 133). A sex-stratified diagnostic subgroup analysis showed that the male-specific effects were again driven by the EOS group. No significant age-by-diagnosis interactions were found for FA, MD, RD, and AD (Supplementary Table S13).

### Associations with clinical measures in adolescents with EOP

After correction for multiple comparisons, we found no significant associations between current medication use, CPZ, and tract-specific FA, MD, RD or AD in adolescents with EOP (whole sample: antipsychotics user = 256, non-user = 29; Lithium user = 27, non-user = 247; antidepressants user = 57, non-user = 171; antiepileptics user = 14, non-user = 257, CPZ = 234; see Supplementary Table S14). Similarly, no DTI measures were significantly associated with symptom severity measures (*n* = 249, PANSS negative/positive). However, longer duration of illness was associated with significantly lower MD in the ALIC and IC (*p* ≤ 0.002, see Supplementary Fig. 10). Lower average RD and AD of BCC and CC were also associated with longer duration of illness (*p* ≤ 0.002). Furthermore, higher AD of the ALIC was associated with later age at illness onset (see Supplementary Fig. 11).

### Comparison to adult individuals with schizophrenia

**Fig. 4:**
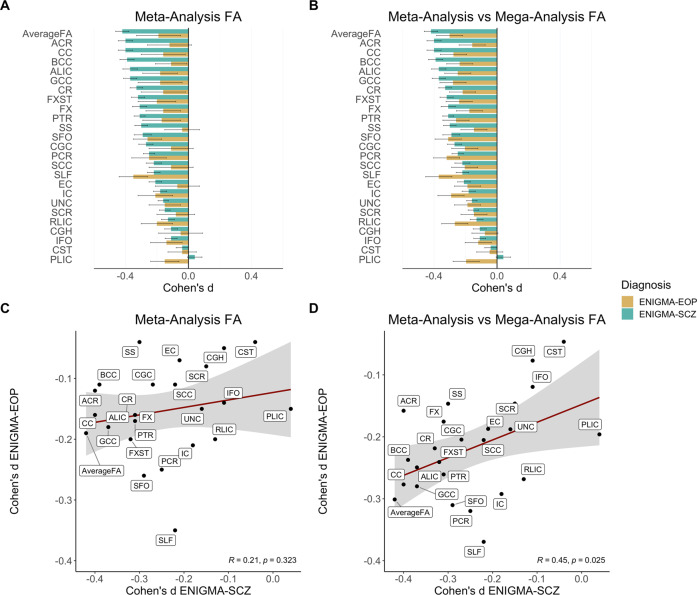
Cohen’s *d* effect sizes in early-onset psychosis (EOP) and adult schizophrenia (SCZ) relative to healthy controls from a prior publication^1^. **A** Meta-analytically derived effect sizes for tract-specific fractional anisotropy (FA) in EOP relative to healthy controls and in adult schizophrenia relative to healthy controls. **B** Mega-analytically derived effect sizes for tract-specific FA in EOP relative to healthy controls and meta-analytically derived effect sizes in adult schizophrenia relative to healthy controls. **C** Correlation in meta-analytically derived effect sizes of tract-specific FA between EOP and adult schizophrenia. **D** Correlation in mega- and meta-analytically derived effect sizes of tract-specific FA between EOP and adult schizophrenia, respectively. In both EOP and SCZ, meta-analytic results were adjusted for age, sex, and linear and nonlinear age and sex interactions (age-by-sex interaction, age [2], and age [2]-by-sex interaction). Cohen’s *d* values and their standard errors are displayed. SK provided values for SCZ.

Differences in the magnitude of tract-specific effect sizes between EOP and adult schizophrenia were observed, with effects being generally less pronounced in EOP (Fig. 4a, b). We observed larger effect sizes for FA in EOP relative to adult schizophrenia only for the SLF and IC (including PLIC and RLIC), but these differences were not statistically significant (see Supplementary Table S18).

Mega-analytically derived Cohen’s *d* values for tract-specific FA in EOP vs. healthy controls were significantly correlated with the meta-analytically derived values in adult schizophrenia vs. healthy controls (r = 0.45, *p* = 0.026). In contrast, the same effect sizes in adult schizophrenia were not correlated with the meta-analytically derived Cohen’s d values in EOP (r = 0.21, *p* = 0.324). Scatterplots for the effect size correlation are displayed in Fig. 4c, d.

## Discussion

We found widespread lower FA in individuals with EOP compared to healthy controls, with the largest effect sizes in the superior longitudinal fasciculus (SLF, *d* = 0.37), posterior corona radiata (PCR, *d* = 0.32), and superior fronto‐occipital fasciculus (SFO, *d* = 0.31). Regions of the corpus callosum (CC), internal capsule (IC), and posterior thalamic radiation (PTR) also showed significant effects with Cohen’s *d* > 0.25. Lower FA in EOP was accompanied by widespread higher RD, and more localized higher MD of the fornix (FX) and uncinate fasciculus (UNC). Higher AD for EOP individuals was observed in the FX. Case-control differences in brain white matter microstructure were driven by individuals diagnosed with EOS, who represent the majority of the EOP sample.

The largest effect for lower FA in EOP was observed for the SLF. This finding is in contrast to effects previously reported in adult samples [1], where the largest effects were observed for average FA, anterior corona radiata, and corpus callosum, suggesting that differences in the SLF may be more pronounced in early-onset populations. The SLF is a major association tract connecting the parietal and temporal lobes with the frontal cortex, and has been implicated in working memory, attention, language, and emotion processing [40]. Significant deficits in SLF white matter microstructure have previously been reported in youth with subsyndromal psychotic-like symptoms [41], clinical high-risk groups [42], EOP [22], and in recently diagnosed schizophrenia [43]. These findings suggest that the SLF may play a role in the development of psychosis. Similarly, lower FA in the PCR and corpus callosum (CC) has also been reported in clinical high-risk populations [42, 44, 45]. In addition, differences in white matter of the SFO, PCR, and CC have been associated with the transition to psychosis [46]. Similar to other major white matter tracts, FA within the SLF increases significantly during adolescence [47]. Interestingly, FA of the SLF showed a positive association with working memory performance in healthy individuals and individuals with EOP [43], and may partially mediate increases in verbal fluency as a function of increasing age [47]. Hence, FA deficits in the SLF may contribute to cognitive disturbances commonly reported in psychotic disorders.

A complementary meta-analysis of FA differences corroborated the significant effect of lower FA in the SLF. Yet, similar to Gurholt et al. [15], the meta-analysis suggests a great degree of heterogeneity across the included samples, likely reflecting differences in inclusion procedures (Table S2), imaging sequences (Table S3), and the inherent clinical diversity of EOP. Furthermore, not all samples from the mega-analysis could be included in the meta-analysis due to sample size limitations (*n* < 10), resulting in a higher variance between tracts and diagnostic groups in the meta- relative to the mega-analysis (see Fig. 4).

In the present study, no regional FA differences remained significant after covarying for global effects. However, lower FA in the SLF was still significant after adjusting for periphery FA, suggesting that the effects observed in the SLF are being driven by differences in core white matter, as opposed to periphery white matter. Overall, the findings suggest that lower FA across the entire white matter skeleton is driving the difference in FA across almost all ROIs. Similar effects were observed for MD, RD, and AD. These findings reflect a pattern of globally lower DTI measures, commonly observed in adults with schizophrenia [1]. While the signature of widespread FA deficits in EOP appears similar to that of adult schizophrenia, notable differences in the magnitude of tract-specific effect sizes between EOP and adult schizophrenia were observed, with effects being generally less pronounced and more variable in EOP (see Fig. 4). In adult schizophrenia, lower FA was most prominent in the anterior corona radiata (*d* = 0.4) and CC (*d* = 0.39) [1]. In EOP, effect sizes were largest for the SLF (*d* = 0.37), PCR (*d* = 0.32), and SFO (*d* = 0.31). Furthermore, FA differences in the SLF remained significant after controlling for periphery FA, unlike the effects observed in adult samples that were found to be driven by peripheral regions [1]. Larger effect sizes for FA in EOP relative to adult schizophrenia were found for the SLF and internal capsule (posterior/retrolenticular limb). Yet, using z tests, these differences in effect sizes between adolescent EOP and adult schizophrenia were not statistically significant. Furthermore, our cross-diagnostic correlation analysis indicated significant convergence with white matter tracts predominantly affected in adolescent EOP relative to adult schizophrenia. However, mega-analytically, not meta-analytically, derived effect sizes in EOP were significantly correlated with the meta-analytically derived values in adult schizophrenia. The discrepancy in the results is likely driven by the lower number of sites included in the meta-analysis. Four sites with less than 10 participants per diagnostic group were excluded (see Supplementary Note S3).

The diagnostic subgroup analysis showed that widespread lower FA was limited to EOS. No case-control differences in FA were found for AFP and other psychosis. This finding is not in line with previous studies in adolescents [48, 49] and adult affective psychosis [50], reporting lower FA in different white matter tracts. Inconsistencies with previous results may be explained by the unbalanced sizes of the subgroups and differences in subgroup characteristics such as age of onset, disease duration, and psychotic features (Table 3). Larger samples are needed to stratify for clinical subgroups in relation to white matter structure.

No significant age-by-diagnosis interactions were observed. However, MD in the ALIC and IC were negatively associated with duration of illness. Similarly, average RD and AD in the BCC and CC were negatively associated with the duration of illness. Furthermore, AD in the ALIC was positively associated with age of onset. These findings suggest that the white matter differences in these regions may be linked to disease progression, as opposed to developmental factors. However, as EOS is associated with a longer duration of illness, these findings may also reflect an effect of the diagnostic subgroup.

We found no significant sex-by-diagnosis interaction for DTI measures, except for RD in the SCC. However, a sex-disaggregated analysis showed that male adolescents with EOP had widespread lower FA relative to healthy male controls, whereas females with EOP did not significantly differ from healthy female controls. Plotting average FA by sex and diagnostic group against age further highlighted consistently lower FA values in male individuals with EOP compared to healthy male controls across the studied age range (Fig. 3B). Average FA in female individuals with EOP did not differ from female healthy controls between the ages of 12 to 18 years. This finding suggests more pronounced white matter alterations in male individuals with EOP relative to same-sex healthy controls and female individuals with EOP. Sex differences in the developmental trajectory of white matter have been reported, with males typically showing protracted white matter maturation compared to females [20, 51, 52]. As these differences may correspond to the impact of sex hormones on white matter during pubertal maturation [53], our findings may reflect the potential protective effect of estrogen for females against development and severity of psychosis [54]. However, longitudinal studies are needed to establish whether white matter maturation differs between the sexes in EOP relative to healthy same-sex controls.

There were no significant differences between medication users and non-users and impact of CPZ on white matter microstructure, which is similar to findings observed in adult schizophrenia [1]. In addition, no significant associations between white matter and symptom severity were found, also in agreement with previous findings in EOP [2] and adult samples [1].

This study is subject to some limitations. Firstly, the cross-sectional design of this study does not allow for a more thorough investigation of the effects of sex, duration of illness, and medication exposure. Secondly, FA is a summary measure of white matter microstructure that does not map perfectly onto the microstructural properties of the underlying tissue. The observed differences in FA may be influenced by a number of neurobiological processes, including changes in fiber organization such as packing density and axon branching, as well as alterations in myelination [16, 55, 56]. In this study, we found that lower FA largely overlapped with higher RD, indicative of either demyelination or dysmyelination [16], with minimal changes to AD. However, inflammatory processes associated with psychosis onset can also impact DTI measures [57–59]. Advanced imaging techniques, such as free-water imaging, separates the contribution of extracellular water from water diffusing along the axon to allow for improved specificity to detect microstructural differences [60, 61]. When considering our findings, it is also important to account for the potential impact of ongoing white matter maturation in adolescent EOP, e.g., FA increases are typically reported throughout childhood and early adulthood. However, studies of white matter maturation in EOP have reported inconsistent findings, suggesting diverging, converging, or parallel developmental trajectories to healthy individuals [2].

Finally, tract-based spatial statistics (TBSS) is commonly used to perform voxel-based analysis of white matter [62]. However, the method is not without limitations. For example, spatial normalization can result in misalignment, with smaller tracts being particularly susceptible [63]. In addition, smaller atlas ROIs such as the FX and CST are more vulnerable to partial volume effects and motion artifacts. Nevertheless, the ENIGMA-DTI Working Group has rigorously tested the reproducibility of measures using this TBSS approach for ROI analyses [64]. Further, we combined neuroimaging datasets from nine sites, introducing heterogeneity due to different scanners, vendors, and sequences. In line with recommendations from the ENIGMA consortium, we addressed this issue by using the batch adjustment method, ComBat, which has been shown to reduce site-related heterogeneity and to increase statistical power [35]. However, residual scanner effects may still be present. Similarly, motion is a confound in diffusion weighted imaging studies [32]. Although we found no case-control differences in average motion relative to the first volume and motion correction was performed during preprocessing, unaccounted motion effects may influence the results.

In the largest analysis of white matter differences in EOP to date, we found widespread lower FA and higher RD with more localized differences in MD and AD for EOP relative to healthy controls. In contrast to what has previously been reported in adult samples, the largest effects for EOP were observed in the SLF and PCR, followed by interhemispheric and thalamo-cortical regions. Differences were most pronounced in male individuals with EOS relative to same-sex healthy controls. The global pattern of widespread microstructural alterations observed in EOP solidifies the hypothesis that schizophrenia may be a disorder of global brain structural connectivity. Future analyses of longitudinal data will allow for a more in-depth investigation of brain maturation in EOP and for further explorations of the effects of sex, duration of illness, and medication exposure.

## Supplementary information

Supplemental Material
